# Applications of Near-Infrared Spectroscopy for Nondestructive Quality Analysis of Fish and Fishery Products

**DOI:** 10.3390/foods13243992

**Published:** 2024-12-10

**Authors:** Jiaojiao Zhou, Chen Liu, Yujun Zhong, Zhihui Luo

**Affiliations:** 1 National R&D Center for Se-Rich Agricultural Products Processing, School of Modern Industry for Selenium Science and Engineering, Wuhan Polytechnic University, Wuhan 430023, China; jiaojiaozhou@whpu.edu.cn; 2 School of Food Science and Engineering, Wuhan Polytechnic University, Wuhan 430023, China; chen.liu@whpu.edu.cn; 3 Guangxi Key Lab of Agricultural Resources Chemistry and Biotechnology, College of Chemistry and Food Science, Yulin Normal University, Yulin 537000, China; 18778837076@163.com

**Keywords:** near-infrared reflectance spectroscopy (NIRS), fish, fishery products, partial least squares (PLS)

## Abstract

Fish has become one of the most popular aquatic products for its beneficial effects. The quality of fish and fishery products may be influenced by their geographical origin, transportation, processing, and storage conditions. The availability of rapid and reliable techniques is important for nondestructive determination of their quality. Recently, near-infrared spectroscopy (NIRS) has been widely employed in the nondestructive evaluation of fish and fishery products. However, a comprehensive review on NIRS for this topic remains to be published. Based on this demand, the applications of NIRS in the nondestructive evaluation of fish and fishery products have been discussed in this review. This review firstly introduces the fundamentals of NIRS. Then the application of NIRS for the assessment of species, geographical origin, adulteration, freshness, nutrient components, and texture is summarized. In addition, the application of near-infrared hyperspectral imaging technology in fish and fishery products is also discussed. Finally, the challenges and prospects are outlined. The current review may provide a reference for research on NIRS in this field. In the future, NIRS could be used for online assessment of quality attributes in the fish industry through the development of new instruments and chemometrics.

## 1. Introduction

Fish and its products have become an important part of the human diet owing to their high nutrition and relatively low price. The consumers of fish and fishery products are conscious of their quality. However, fish is very perishable and susceptible, and its quality could be easily influenced by many factors, such as microbial spoilage and chemical contamination. Meanwhile, consumers are becoming more conscious of quality. Therefore, apart from a series of preservation methods such as freezing [1], cooling [2], drying [3], salting [4], smoking [5], and fermentation [6] to maintain quality, novel evaluation tools are clearly significant.

Until now, fish quality issues can be assessed by sensory evaluation, gas chromatography mass spectrometry (GC-MS), high-performance liquid chromatography (HPLC), near-infrared spectroscopy (NIRS), Raman spectroscopy, etc. Generally, sensory evaluation offers the best quality information to consumers due to its visibility [7]. But despite its general acceptance, it still has limitations in fish processing. Therefore, chemical and physical analytical methods are also used to assess the quality of fish and fishery products. Meanwhile, sensory evaluation results should be consistent with instrumental results. The physical properties of fish and fishery products include rigor mortis, color attributes, and textural features, which can be assessed through morphological analysis [8]. Chemical analytical methods are usually associated with changes in chemical composition such as protein decomposition, lipid oxidation, and microbial spoilage. Conventional methods, such as the use of a pH meter for pH determination, HPLC for K value determination, and the semimicro Kjeldahl method for total volatile basic nitrogen (TVB-N) determination, are commonly used for freshness evaluation of fish and fishery products. Meanwhile, the analytical results of these methods are generally regarded as reference values to establish corresponding prediction models. 

With the development of the industry, rapid analytical methods have become an imperative demand. Near-infrared spectroscopy (NIRS) stands out due to its rapid analytical speed, equal or superior to that of reference methods, the opportunity to conduct both qualitative and quantitative tests, the low cost per test, and the ability to make non-invasive measurements. NIRS has been widely used for the assessment of species, nutrients, freshness, authenticity, and geographical origin in the fish industry [9,10,11,12]. There are several reviews on the quality evaluation of cocoa beans and cocoa bean products [13], stored grains [14], dairy production [15], oilseeds and edible oils [16], tea [17], and fish muscle [18], as well as the evaluation of the chemical, textural, and structural characteristics of meat [19]. However, there have been no reports on the application of chemometrics to NIR and HSI spectral data for evaluating the quality of fish and fishery products. Accordingly, this review summarizes the recent developments of fish and fishery products based on NIRS and HSI technique in tandem with chemometrics.

## 2. Fundamentals of NIRS 

**Figure 1:**
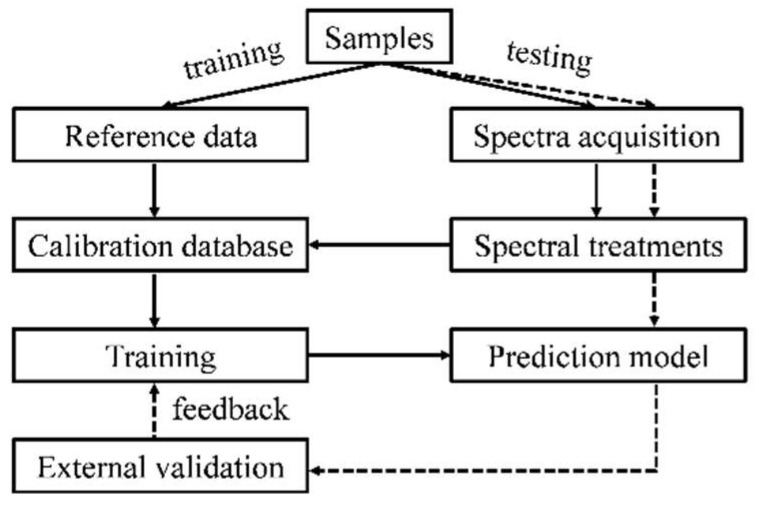
Generalized workflow of near-infrared spectroscopic modeling procedures. Continuous arrows: training process of multivariate calibration; dashed arrows: testing of the trained models with validation.

The NIR spectral region was discovered by William Herschel in 1800 [20]. NIR light covers an electromagnetic spectrum (approximately 780–2500 nm). NIRS is a widely used spectroscopic method, which shines NIR light on a sample and records the reflected or transmitted radiation [15]. NIRS involves a combination of overtones or basic absorption bands associated with molecular rotation and vibrational transitions [21]. Information about the characteristics of chemical bonds can be obtained via scanning the near-infrared spectrum of the sample [22]. However, there are many factors that influence NIR light absorbance. Thus, it is difficult to directly analyze the recorded NIR spectra based on wavelength and related absorbance. Meanwhile, each substance, such as grains, fruits, milk, or dairy products, has its unique NIR spectrum. Therefore, NIRS could be used for qualitative and quantitative analysis. A set of representative spectra of the test samples is required to determine its quality. In fact, NIRS is an indirect measurement, and reference measurements are essential in most cases. The commonly used method is to take both spectral information and reference measurements in a training set to establish a prediction model, which is then validated on further samples and makes predictions about unknown samples based on the validated model. A training set with both reference data and spectra can be used to establish quantitative calibration or qualitative classification models. The characteristics of samples that are similar in nature may be predicted using the trained model in a validation set. The comparison between predicted values and reference values may provide feedback on the effectiveness of the established prediction model (Figure 1). To be noted, NIR spectra contain different types of background interference. Spectral preprocessing is necessary to reduce noise, signal distorting effects, and other interfering factors. In data processing, the properties of the dataset and the research objectives will determine the form of data preprocessing and the required chemometrics. The chemometric methods employed to calibrate models include partial least squares (PLS) regression, principal components analysis (PCA), artificial neural networks (ANN), and support vector machines (SVM) [16]. Specifically, PLS is a procedure used to reduce original variables to a new variable set based on a small number of orthogonal factors. 

## 3. Applications in Fish and Fish Products Quality and Safety Analysis 

### 3.1. Qualitative Analysis

Fish authentication is a major concern to stakeholders. The substitution of high-priced fish species with cheap substitutes is an economic deception, and could also affect consumer health [23]. Traditional methods are ineffective at identifying adulterated fish and fish products due to such products’ similar physical and chemical attributes. Therefore, it is proposed to employ NIRS for the identification of adulteration and authentication of fish and fish products. Their qualitative analysis mainly focuses on two aspects: variety and geographical origin. Table 1 presents applications of NIRS in identifying adulteration and authenticating fish and fish products. 

NIRS has been successfully used in distinguishing fresh and frozen/thawed fillets [24,25,26,27], organic and conventionally farmed European sea bass [28], wild and farmed European sea bass [29], raw and cooked freeze-dried rainbow trout [30], and fillets and patties of Atlantic cod from those of haddock [31]. The classification accuracies range from 65% to 100%. Different species of fish fillets with the same geographical origin could also be identified by vis-NIR combined with principal component analysis (PCA). Qin et al. used vis-NIR coupled with 24 machine-learning classifiers to distinguish fish fillets of six species, including red snapper, vermilion snapper, Malabar snapper, summer flounder, white bass, and tilapia [23]. The overall classification accuracy for species differentiation is 98.5%. However, it is difficult to differentiate adulterated fish or fish products from those of cheaper fish or fish products, or to detect the mislabeling of fish fillets.

In addition, the geographical origin of fish and fishery products will also influence their quality. NIRS was utilized to discriminate fish meal produced in China and Peru with good accuracy and precision [32], which provided a precise tool to identify commercial fish meal products. The discrimination accuracy for the validation set increased to 94.87% after the selection of characteristic wavelength variables by competitive adaptive reweighted sampling (CARS). The principle of the CARS algorithm is that through adaptive reweighted sampling (ARS), a PLS model is established based on the new subset using the wavenumbers with a greater absolute weight of the regression coefficients. After multiple calculations, the wavenumbers in the subset with the smallest root mean square error (RMSECV) were selected [33]. Therefore, the CARS algorithm could be used to extract the optimal variables. Meanwhile, NIR reflectance spectroscopy was employed to authenticate European sea bass from different fishing areas [34]. Briefly, orthogonal partial least square discriminant analysis (OPLS-DA) was used to develop classifiers to discriminate fish samples. As a result, the samples were predicted with good accuracy. Soft independent modelling of class analogy (SIMCA) includes a first step of independent PCA applied to spectral variables of the calibration sets for the two considered classes [35]. Then, for each class, the number of principal components with a minimum threshold of 90% explained variance is chosen and the standard deviation of the residuals is calculated. Liu et al. investigated the combination of NIRS and SIMCA to assess the origin traceability of tilapia fillets and reported good precision [36]. Similarly, NIR reflectance spectroscopy coupled with PLS was used to discriminate salted ripened anchovies from different fishing areas [9]. The discrimination models exhibited good prediction precision. From the above applications, we can conclude that NIRS can effectively discriminate fish and fish products from different geographical origins. 

**Table 1:** Summary of application of NIRS in identifying adulteration of fish and fish products.

Samples	Applications	Spectral Range (nm)	Models	Accuracies	References
West African Goatfish	Classification	300~1100	PLS	100%	[24]
Fresh and frozen/thawed fish	Classification	1100~2500	PLS	80~91%	[25]
Fresh and frozen/thawed tuna fillets	Classification	350~2500	PLS	>82%	[26]
European sea bass	Classification	1100~2500	PCA + SIMCA	90%	[28]
European seabass	Classification	1100~2500	PLS		[29]
Atlantic cod	Classification	950~1650	LDA + SIMCA	100%	[31]
Seven freshwater fish species	Classification	1000~1799	PCA + LDA	100%	[37]
Fish meal	Classification	1000–2500	PLS-DA + CARS	94.87%	[32]
European sea bass	Geographical origins	1100–2500	PCA + OPLS-DA	85–100%	[34]
Tilapia fillets	Geographical origins	1000–2500	SIMCA	75%	[36]
Salted ripened anchovies	Geographical origins	1000–2400	OPLS-DA	99%	[9]

### 3.2. Quantitative Quality Analysis of Fish and Fishery Products 

Recently, NIRS coupled with chemometrics has been successfully applied to the evaluation of chemical properties, such as nutritional composition and freshness index. An overview of quantitative quality analysis of fish and fishery products using NIRS is listed in Table 2. 

Free fatty acids and moisture in fish oils were determined with partial least squares regression (PLSR) analysis by means of NIRS [38]. Likewise, the fatty acids and lipid classes in salmon oil were studied by NIRS combined with PLSR. The results indicated RMSEP values between 0.29 and 1.78% and residual predictive deviation (RPD) values in the range of 1.27 and 6.44 [39]. NIRS was also successfully used to monitor sodium chloride during the cod fish desalting process [40]. These studies clearly demonstrated that NIRS can be successfully used for inspection of the chemical composition of fish and fishery products. 

Freshness is an important quality parameter of fishery products. The combination of spectral and freshness features has been reported in applications related to the grading of fishery products and differentiation between fresh and frozen/thawed fillets [41]. Trimethylamine (TMA) is another quality indicator for assessing fish freshness [42]. A genetic algorithm PLS (GA-PLS) model was established using FT-NIR for quantitative prediction of TMA in fish [43]. Several different PLSR models were compared using FT-NIR for quantification of TMA. Among the established calibration models, the GA-PLS achieved the best results with R_p_^2^ of 0.977 and RMSEP of 5.10. As a kind of nitrogen-containing compound, TVB-N is also an indicator of fish freshness. TVB-N value has been determined by NIRS with a back-propagation artificial neural network (BP-ANN) as well as a computer visualization of *Parabramis pekinensis* samples purchased from a local market. Acceptable prediction results were obtained with a discrimination rate of 93.33% in the prediction set [44]. Microbial spoilage is another factor that shortens the shelf life for fishery products. NIRS has been applied to estimate the microbial spoilage on Atlantic salmon [45]. A PLSR calibration model achieved good prediction results with R_CV_^2^ and RMSECV of 0.95 and 0.12, respectively. Different from the usually exploited PLS, excellent results were obtained using a combination of GA and BP-ANN for the evaluation of total bacteria in flounder fillet [46], and the R_p_^2^ and RMSEP were calculated as 0.985 and 0.095, respectively. Histamine levels in frozen/thawed tuna were also measured by NIRS with PLSR as well as machine learning. Good predictive performance was obtained with R_p_^2^ of 0.74 and R_CV_^2^ of 0.888 [47]. Similarly, Pauline et al. used an optimized wavelet neural network (WNN) model to predict the histamine levels in mackerel with acceptable results [48]. The K value is also frequently used for freshness evaluation and represents adenosine triphosphate (ATP) decomposition [49]. Ding et al. found that NIRS together with SVM model outperformed PLS-related models in predicting the K value in silver chub, with R_p_^2^ of 0.9374 and RMSEP of 0.036525 [50]. In another study, modeling of the K value in silver carp pieces using NIRS was also satisfactory [51]. The performance of the established PLS model showed better K value prediction ability compared with other chemometric algorithms, with R_p_^2^ of 0.9786 and RMSEP of 3.98. Apart from individual indicators, simultaneous prediction multiple freshness indicators have also been reported based on NIRS and PLSR calibration analysis [52,53]. These results indicate that NIRS combined with chemometrics has great capability for freshness assessment.

NIRS with chemometric analysis has also been developed to determine the textural properties of fish fillets. For instance, Zhou et al. revealed the feasibility of NIRS based on PLSR models to predict the textural properties of silver carp flesh [54]. Based on full spectral analysis, the established PLSR models displayed less successful performance. To reduce redundancy, key wavelengths were chosen, and R_P_^2^ values between 0.83 and 0.95 with the corresponding RMSEP values ranging from 0.08 and 2.63 were obtained. The statistical results indicated that NIRS could assess the textural properties of fish.

Overall, these results indicate that NIRS combined with chemometrics for the prediction of the physicochemical properties and quality of fish and fishery products is feasible.

**Table 2:** Overview of application of NIRS for quantitative quality analysis of fish and fishery products.

Samples	Parameters	Spectral Range (nm)	Model	Performance	Ref.
Silver carp	trimethylamine	1000~2500	PLS	R^2^_P_: 0.977, RMSEP: 5.10	[43]
*Parabramis pekinensis*	TVB-N	1000~2500	ANN	Accuracy: 0.9333	[44]
Atlantic salmon	microbial numbers	800~2500	PLS	R^2^_P_: 0.95, RMSEP: 0.12	[45]
Flounder fillets	total bacteria	600~1100	ANN	R^2^_P_: 0.966, RMSEP: 0.083	[46]
Tuna fish	histamine	400~2500	PLS	R^2^_P_: 0.74, R^2^_cv_: 0.88	[47]
Mackerel	histamine	400~2498	WNN	R^2^_P_: 0.79, RMSEP: 70	[48]
Silver chub	*K* value	1000~2500	SVM	R^2^_P_: 0.9374, RMSEP: 0.036525	[50]
Silver carp	*K* value	1000~2500	PLS	R^2^_P_: 0.9786, RMSEP: 3.98	[51]
Catfish fillets	TVB-N, thiobarbituric acid (TBA), drip loss, L^*^, and whiteness	1000~2500	PLSR	Accuracy: 0.86–0.90, residual predictive deviation (RPD): 2.66–3.19	[52]
Bighead carp	pH, TVB-N, TBA, and K value	1000~1799	PLSR	R^2^_P_: 0.807–0.954, RMSEP: 0.081–6.509	[53]
Silver carp flesh	textural properties	1000~1799	PLSR	R^2^_P_: 0.83–0.95, RMSEP: 0.08–2.63	[54]

## 4. Near-Infrared Hyperspectral Imaging Technology in Fish and Fishery Products

NIR hyperspectral imaging (NIR-HSI) technology is a spatially resolved spectral technology that utilizes the advantages of spectroscopy and imaging technology [55]. As an alternative to traditional methods, it has been widely used in food quality and safety analysis [56]. A summary of its applications in fish and fishery products is presented in Table 3. 

Many studies have reported the application of NIR-HSI for the identification of fraud and mislabeling in fish fillets [23], for the classification of fresh and frozen/thawed fish fillets [57,58,59], for the automated sorting of Atlantic cod roe, milt, and liver [60], for the automatic differentiation of organic and conventional salmon fillets [61], and for the investigation of mass changes of vacuum freeze-dried grass carp fillets [62]. These successful applications have proved that using NIR-HSI to qualitatively predict the quality attributes in fish industry is feasible.

NIR-HSI techniques have been widely used to predict the chemical properties of fish and fishery products, such as moisture and lipid content, total volatile basic nitrogen, K index value, and microbial spoilage. For example, He et al. used NIR-HSI to predict the moisture distribution, drip loss, and pH value of salmon fillets [63,64]. Moreover, it has been also used for the determination of fish caloric density. Xu et al. used this technique for accurate determination of the gross energy density values of salmon fillets [65]. Good results were obtained, with R^2^_P_ of 0.908 and RMSEP of 6.871%. Subsequently, Qu et al. proved the feasibility of NIR-HSI to determine moisture contents in grass carp slices [66]. In another work, Anderssen et al. used this technique to predict liquid loss in frozen and thawed cod and further proved its feasibility [67]. Zhang et al. measured fat and moisture content in salmon fillets with an SVM model based on characteristic wavelengths [68]. Similarly, Aurora Lintvedt et al. measured the fatty acids in salmon fillets using NIR-HSI, with R^2^ ranging from 0.831 to 0.92 [69]. Thus, chemometrics applied to NIR-HSI can be used for the prediction of chemical information. In terms of freshness measurement of fish, NIR-HSI has been successfully applied to establish a prediction model of the total volatile basic nitrogen (TVB-N) content in fish fillets [70,71], the shelf life of expired vacuum-packed smoked salmon [72,73], the determination of *K* value [74], and the evaluation of microbial spoilage [75,76,77,78]. 

NIR-HSI techniques are also available for evaluating the physical attributes of fish [79]. Images were taken with a least square support vector machine (LS-SVM) to assess the tenderness of raw farmed salmon fillets [80]. After wavelength selection by a successful projections algorithm (SPA), an R^2^_P_ value of 0.905 and RMSEP of 1.089 were obtained for the optimized SPA-LS-SVM prediction model. Cheng revealed that LS-SVM has the ability to evaluate the firmness of grass carp fillets [81]. After selecting the important wavelengths by GA, the best performance showed an R^2^_P_ of 0.941 and the RMSEP was 1.229. Therefore, NIR-HSI combined with GA-LS-SVM could detect the firmness of grass carp fillets. Color is another physical parameter in the quality evaluation of fillet. Color distribution measurements using NIR-HSI are also available for salmon fillets based on multiple linear regression (MLR) models [82]. Similarly, Wang et al. have successfully applied NIR-HSI to monitor the color changes of large yellow croaker fillets during low-temperature storage using a PLSR model [83]. These results indicate that NIR-HSI can be used as a non-invasive and rapid alternative to traditional colorimeters in color measurements of fish fillets.

**Table 3:** A summary of application of NIR-HSI technology in fish and fishery products.

Samples	Parameters	Spectral Range (nm)	Model	Performance	Ref.
Fish fillets	inspect substitution and mislabeling	400~1000 and 900~1700	SVM; SVM	Accuracy: 95.0% and 95.5%	[23]
Halibut fillets	fresh and frozen/thawed samples	380~1030	SVM	Accuracy: 97.22%	[57]
Grass carp fillets	fresh and frozen/thawed samples	400~1000	SVM	Accuracy: 94.29%	[58]
Crucian carp	fresh and froze/thawed samples	400~1000	PLS	Accuracy: over 92.5%	[59]
Atlantic cod	roe, milt, and liver	400~1000	Spectral Angle Mapper (SAM)	Sensitivity: 96%; specificity: 98%	[60]
Salmon fillets	organic and conventional salmon fillets	400~1000	SVM	Accuracy: 98.2%	[61]
Dried grass carp fillets	dehydrating and rehydrating mass changes	400~1000	PLSR	R^2^_P_: 0.8278, RMSEP: 9.79%	[62]
Salmon fillets	moisture	400~1000; 900~1700; 400~1700	PLSR	R^2^_P_: 0.893, RMSEP: 1.513%; R^2^_P_: 0.902, RMSEP: 1.450%; R^2^_P_: 0.849, RMSEP: 1.800%;	[63]
Salmon fillets	drip loss and pH distribution	400~1700	PLSR	R^2^_P_: 0.834, RMSEP: 0.067 (drip loss); R^2^_P_: 0.877, RMSEP: 0.046 (pH)	[64]
Salmon fillets	gross energy density	900~1700	PLSR	R^2^_P_: 0.908, RMSEP: 6.871%	[65]
Grass carp slices	moisture	400~1000	PLSR	R^2^_P_: 0.9021, RMSEP: 0.0561	[66]
Frozen and thawed cod	liquid loss	430~1000	PLS	R^2^_P_: 0.88, RMSEP: 0.0062	[67]
Salmon fillets	fat and moisture	900~1700	LS-SVM	R^2^_P_: 0.9685, RMSEP: 1.1750 (fat); R^2^_P_: 0.9688, RMSEP: 0.8021 (moisture)	[68]
Salmon fillets	fatty acids	930~2500	PLSR	R^2^_P_: 0.9, RMSEP: 0.86	[69]
Tilapia fillet	TVB-N	900~1700	PLSR	R^2^_P_: 0.8524, RMSEP: 2.4487	[70]
Rainbow trout fillets	TVB-N	430~1010	Linear Deep Neural Network (LDNN)	R^2^_P_: 0.853, RMSEP: 3.159	[71]
Vacuum-packed smoked salmon	shelf life	400~1000	PLS	R^2^_P_: 0.97, RMSEP: 0.08	[72]
Vacuum-packed chilled smoked salmon	shelf life	400~1000	support vector machine (SVM)	R^2^_P_: 0.89, RMSECV: 7.7	[73]
Grass carp and silver carp fillets	*K* value	400~1000	SVM	R^2^_P_: 0.936, RMSEP: 5.21%	[74]
Salmon flesh	total viable counts	400~1700	PLSR	R^2^_P_: 0.985, residual predictive deviation (RPD): 5.127	[75]
Salmon flesh	lactic acid bacteria	900~1700	SVM	R^2^_P_: 0.925, RMSEP: 0.531	[76]
Salmon flesh	total counts of *Enterobacteriaceae* and *Pseudomonas* spp. (EPC)	900~1700	PLS	R^2^_P_: 0.964, RMSEP: 0.429	[77]
Grass carp fillets	total viable counts	400~1000	PLSR	R^2^_P_: 0.90, RMSEP: 0.57	[78]
Salmon fillets	tenderness	400~1720	SVM	R^2^_P_: 0.905, RMSEP: 1.089	[80]
Grass carp fillets	firmness	400~1000	SVM	R^2^_P_: 0.941, RMSEP: 1.229	[81]
Salmon fillets	color	964~1631	MLR	R^2^_P_: 0.876, 0.744, and 0.803 for L*, a*, and b*, respectively.	[82]
Large yellow croaker fillets	color	400~1000	PLSR	R^2^_P_: 0.908, 0.915, and 0.977 for L*, a*, and b*, respectively.	[83]

## 5. Conclusions and Future Trends

In summary, NIRS combined with a chemometric algorithm has been proved to be an effective tool for nondestructive and rapid quality analysis in the fishery industry. The application of NIRS to fish and fishery products will greatly benefit the quality and safety of the industry. However, there are still some limitations affecting the reliability and accuracy of prediction models based on NIRS. Firstly, prediction models based on NIRS require abundant sample analysis as reference results. Moreover, spectral information is complicated and largely relies on analytical methods. For instance, improper wavelength selection may leave out useful information or retain useless information in the spectra. More attention should be paid to developing more advanced algorithms. Second, sample preparation methods will influence the performance of prediction models, especially in terms of the reference value determination of samples. Third, veterinary drug residues and heavy metal ions are also hazards in the fishery industry, and innovative methods should be established to deal with these contaminants in the future. Finally, online measurement in the food industry is another direction to be explored in the future.

## Data Availability

No new data were created or analyzed in this study.
